# The Gut Microbiota–Metabolic Axis: Emerging Insights from Human and Experimental Studies on Type 2 Diabetes Mellitus—A Narrative Review

**DOI:** 10.3390/medicina61112017

**Published:** 2025-11-11

**Authors:** Mohammed Saad Alqahtani

**Affiliations:** Department of Internal Medicine, College of Medicine, Prince Sattam Bin Abdulaziz University, Al-Kharj 11942, Saudi Arabia; ms.alqahtani@psau.edu.sa

**Keywords:** gut microbiota, metabolic axis, type 2 diabetes mellitus, narrative review

## Abstract

The rapidly advancing field of gut microbiota research has revealed its pivotal role in human health, with growing evidence implicating microbial dysbiosis in the development of metabolic diseases, particularly type 2 diabetes mellitus (T2DM). This narrative review synthesizes recent findings on the complex, bidirectional relationship between the gut microbiota–metabolic axis and T2DM, drawing upon data from human and experimental studies published in the past decade. Patients with T2DM consistently demonstrate marked gut dysbiosis, characterized by reduced microbial diversity and depletion of beneficial butyrate-producing taxa such as *Faecalibacterium prausnitzii* and *Roseburia intestinalis*. In contrast, increases in pro-inflammatory bacteria including *Escherichia-Shigella* and *Lactobacillus* are commonly observed. Such compositional changes are linked to metabolic dysfunction through altered microbial metabolites, including elevated trimethylamine N-oxide (TMAO), which has been associated with insulin resistance and increased diabetes risk. Moreover, gut microbiota imbalances correlate with systemic inflammation, as indicated by higher levels of cytokines such as IFN-γ and IL-6. These findings underscore the gut microbiota’s central role in energy metabolism and inflammation in T2DM. Understanding these mechanisms could inform novel therapeutic and preventive strategies—such as microbiota-targeted dietary, probiotic, or pharmacologic interventions—to improve metabolic outcomes and enhance clinical management of diabetes.

## 1. Introduction

The human body is home to trillions of microorganisms, such as bacteria, archaea, viruses, and fungi that make up the microbiota [1]. These microbial residents, particularly those within the gut, have a remarkable impact on our health, ranging from the control of digestion to immunity [2]. The story of how our gut microbiota assembles is still unfolding. It is believed by some to begin in utero, while other studies point to birth as the starting mechanism, with the infant gut becoming rapidly colonized and stabilizing sometime between the ages of 2 and 5 [3]. Wherever the beginning, the gut microbiota is a lifelong fellow traveler on our journey of health [2].

The gut is not a uniform milieu. From the small intestine, with its acidic, fast-moving conditions that limit microbial growth, to the large intestine, a seething billions of anaerobic bacteria including *Firmicutes*, *Bacteroides*, and *Actinobacteria*, the microbiota is transformed dramatically [4]. It is this variation, especially in the large intestine, where the microbiota and human host engage in a dynamic dialogue, influencing basic processes like nutrient breakdown, vitamin synthesis, and gut barrier protection [5]. Age, sex, diet, and geography all dictate this microbial population, while prebiotics and probiotics are interventions that can intentionally alter its composition toward health [6,7]. Interestingly, early microbial exposures can even influence the inheritance of disorders like obesity or type 2 diabetes mellitus (T2D) [8,9,10].

T2D, which is hallmarked by chronic hyperglycemia due to insulin resistance and impaired insulin secretion, has surged globally [11]. Between 1990 and 2019, the global incidence of T2D more than doubled from approximately 8.4 million to 21.7 million cases, while diabetes-related deaths increased from about 606,000 to 1.5 million [12]. During this period, the total number of people living with T2D rose from 148 million to 438 million, corresponding to an age-standardized prevalence rate of around 5283 per 100,000 population in 2019 [12]. The burden is particularly high in low- and middle-income countries, including those in the Middle East and South Asia, where rapid urbanization, sedentary lifestyles, and dietary transitions are key contributing factors [13,14].

Gut microbiota dysbiosis, a perturbation of microbial communities, is now firmly implicated as an important driver of T2D development, nurturing systemic inflammation and metabolic dysfunction deteriorating insulin resistance [15,16]. This can lead to severe complications, ranging from diabetic neuropathy to heart diseases. Diabetes mellitus (DM), which includes T2D, is a complex metabolic disorder of multifactorial origin with impaired insulin secretion or sensitivity with raised blood glucose levels [17]. T2D, accounting for 90–95% of DM, is affected by genetic, environmental, and lifestyle factors like obesity, smoking, and low-fiber diets [18,19,20]. Chronic hyperglycemia provokes microvascular disease (e.g., retinopathy, kidney disease) and macrovascular disease (e.g., heart disease), imposing a significant public health burden [21,22].

Recent research has expanded the clinical relevance of this relationship. Emerging evidence links gut microbiota alterations to the modulation of incretin hormones such as GLP-1, which influence glucose homeostasis and may have implications for the course of diabetes during COVID-19 infection [23,24]. Furthermore, incretin-based therapies, including GLP-1 receptor agonists such as semaglutide, have demonstrated not only glycemic benefits but also improvements in quality of life and weight reduction in patients with T2D [25]. Real-world studies evaluating both subcutaneous and oral formulations of semaglutide have underscored these advantages, highlighting their growing importance in clinical management [26,27]. Among the numerous functions of the gut microbiota, its association with T2D is one of the most important areas of investigation. The primary aim of this narrative review was to synthesize emerging evidence on the interplay between the gut microbiota–metabolic axis and the pathophysiology of T2D, highlighting potential mechanisms through which gut microbial alterations may contribute to disease development and progression.

## 2. Materials and Methods

This study was designed as a narrative review aimed at synthesizing current evidence on the relationship between the gut microbiota and the pathogenesis of type 2 diabetes mellitus (T2D) and its associated metabolic disorders. The review was conducted and reported in accordance with the Scale for the Assessment of Narrative Review Articles (SANRA) guidelines, as recommended by the EQUATOR Network for enhancing transparency and scientific rigor [28]. A completed SANRA checklist has been provided as a Supplementary File (Supplemental Table S1). A comprehensive literature search was conducted using PubMed, Scopus, and Google Scholar databases up to January 2025. The search strategy combined keywords and Medical Subject Headings (MeSH) terms relevant to the topic, including gut microbiota, gut microbiome, gut dysbiosis, type 2 diabetes mellitus, insulin resistance, glucose metabolism, metabolites, trimethylamine N-oxide (TMAO), short-chain fatty acids (SCFAs), inflammation, and obesity or cardiovascular diseases. The inclusion criteria encompassed original research articles, systematic reviews, and meta-analyses that investigated compositional and functional alterations of the gut microbiota in relation to T2D. Human studies were prioritized, but relevant animal or mechanistic studies that provided insight into the gut–metabolic axis were also considered. No restrictions were applied regarding publication year, geographic region, or study design to ensure a comprehensive overview of the field. All retrieved studies were screened for relevance by reviewing titles and abstracts, followed by full-text examination. Eligible studies were thematically analyzed, and findings were synthesized narratively, focusing on two key domains: (1) gut microbiota composition changes associated with T2D, and (2) mechanisms linking gut dysbiosis to T2D pathogenesis.

## 3. Summary of Findings

**Figure 1:**
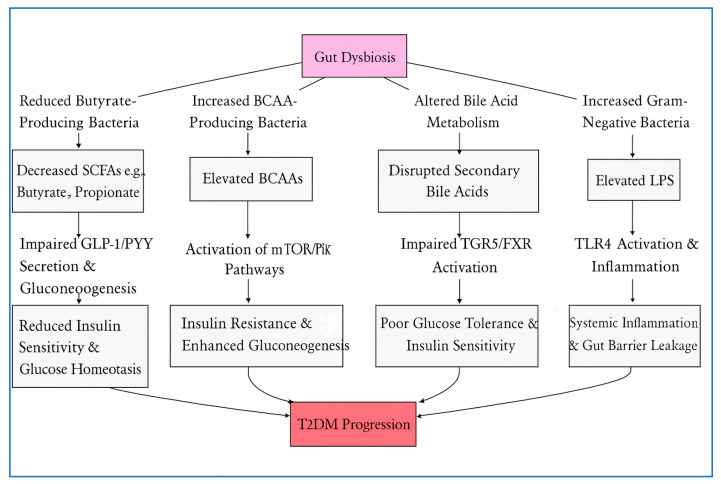
Mechanisms of Gut Microbiota in T2D Pathogenesis.

Our gut bacteria balance is far more important than we once thought, and it plays a critical part in our metabolic health. When this balance is lost, a condition known as dysbiosis can pave the way for Type 2 Diabetes (T2D). This is because a balanced gut microbiome controls our body’s glucose and lipid metabolism, insulin sensitivity, and even systemic inflammation. As shown in Figure 1, these processes are mainly controlled by some key metabolic byproducts, including short-chain fatty acids (SCFAs), branched-chain amino acids (BCAAs), bile acids, and lipopolysaccharides (LPS).

### 3.1. Alterations in Gut Microbiota Composition Associated with Type 2 Diabetes Mellitus

Recent findings point to significant alterations in gut microbiota composition among T2D patients and non-diabetic controls (Table 1). The classic paper by Larsen et al. (2010) showed a marked reduction in the *Clostridia* class and *Firmicutes* phylum of gut microbiota in T2D patients when compared with healthy individuals [29]. There were also positive correlations between plasma glucose concentration and the ratio of *Bacteroidetes* to *Firmicutes* and *Bacteroides*-*Prevotella* to *C. coccoides*-*E. rectale*. Further, increased abundance of *Betaproteobacteria* was observed in T2D patients with impaired glucose tolerance [29]. As disclosed in Table 1, an MGWAS involving 345 Chinese individuals (T2D and non-T2D) by Qin et al. (2012) further disclosed microbial dysbiosis in T2D [30]. T2D patients’ gut microbiota had increased counts of opportunistic pathogens such as *Bacteroides caccae*, *Clostridium hathewayi*, *Clostridium symbiosum*, *Eggerthella lenta*, *Clostridium ramosum*, and *Escherichia coli*. Conversely, butyrate-producing bacteria including *Clostridiales* sp. SS3/4, *Eubacterium rectale*, *Faecalibacterium prausnitzii*, *Roseburia intestinalis*, and *Roseburia inulinivorans* were substantially lower [30]. The same study further recognized a great quantity of mucin-degrading *Akkermansia muciniphila* and sulfate-reducing *Desulfovibrio* species in T2D microbiomes [30].

Similarly, Karlsson et al. (2013) confirmed these results in a European cohort of females diagnosed with T2D, documenting decreased quantity of *Roseburia intestinalis* and *Faecalibacterium prausnitzii* [31]. The researchers also noted higher quantities of four *Lactobacillus* species and reduced quantity of five *Clostridium* species in T2D patients than those patients with normal glucose tolerance. Particularly, *Lactobacillus* abundance was found to be positively associated with HbA1c and fasting glucose levels, however *Clostridium* abundance displayed negative associations with HbA1c, fasting glucose, C-peptide, triglycerides, and insulin levels, signifying a potential role in progression of T2D [31]. Metformin, a commonly prescribed medication for T2D may impact some microbial shifts, which is associated with increased *Escherichia* species and decreased butyrate-producing taxa [32].

A systematic review by Chong et al. (2024) combined evidence from 58 observational studies (2010–2024), and demonstrated similar consistent differences in the composition of gut microbiota between healthy controls and T2D patients [37]. Beta diversity was frequently different, with genera such as *Lactobacillus*, *Escherichia*-*Shigella*, *Enterococcus*, *Subdoligranulum*, and *Fusobacteria* being positively associated with T2D, and *Akkermansia*, *Bifidobacterium*, *Bacteroides*, *Roseburia*, *Faecalibacterium*, and *Prevotella* being negatively associated. Notably, *Escherichia*-*Shigella* exhibited a uniform positive association with T2D, whereas *Faecalibacterium prausnitzii* was potentially protective [37]. Heterogeneity in outcomes and study design of the studies was noted in the review, and emphasized the need for future studies incorporating age-, diet-, and medication-matched controls to establish causality relationships [37].

Also, Letchumanan et al. (2022) reported findings from a systematic review of 18 quantitative epidemiological studies (n = 5489), focusing on gut microbiota in prediabetes (preDM) and newly diagnosed T2D (newDM) than those with normal glucose tolerance (nonDM) [34]. The review found overall lower gut microbial diversity in preDM and newDM. While the makeup of the microbiota varied among studies, four studies found increased *Firmicutes* and decreased *Bacteroidetes* in newDM [34]. At the genus/species level, *Faecalibacterium prausnitzii*, *Roseburia*, *Dialister*, *Flavonifractor*, *Alistipes*, *Haemophilus*, and *Akkermansia muciniphila* were decreased, and *Lactobacillus*, *Streptococcus*, *Escherichia*, *Veillonella*, and *Collinsella* increased in disease groups in a minimum of two studies [34]. *Lactobacillus* was inversely correlated with fasting plasma glucose, HbA1c, and/or HOMA-IR in four studies, pointing to involvement in glucose dysregulation [34]. Dietary factors significantly influenced bacterial abundances and pointed towards the need for further exploration to establish strong associations and explore *Lactobacillus* species/strain-specific effects [34].

Slouha et al. (2024) reported findings from a systematic review to assess gut microbiota changes in T2D patients, evaluating their effect on insulin resistance and metabolic outcomes [35]. The study found *Bacteroides*, *Proteobacteria*, *Firmicutes*, and *Actinobacteria* prevalent in T2D patients and controls but with varying abundance levels [35]. Importantly, *Lactobacillus* spp. and *Faecalibacterium prausnitzii* were substantially found to be reduced in patients with T2D. Increased *Akkermansia muciniphila* was related to higher BMI and decreased lipid metabolism in T2D patients [35]. Metabolites such as butyrates and melatonin were implicated in progression of T2D. Lower levels of hormone testosterone levels in males with T2D correlated was associated with a rise in *Gemella*, *Lachnospiraceae*, and *Massilia* [35]. The review suggests future research should explore how dietary habits, physical activity, and antidiabetic therapy affect composition of microbiota and increased levels of blood glucose [35].

The systematic review by Fatin Umirah et al. took into account the differences in gut microbiota composition between the subjects with T2D and controls [33]. The results showed that the butyrate-producing bacteria were inversely correlated with the glycemic parameters. Second, *Lactobacilli* were also more prevalent in the gut of T2D patients compared to healthy controls, albeit the authors warned that this could be secondary to antihyperglycemic therapy [33]. The review further stated that *Clostridia* and the phylum *Firmicutes* had moderate to high positive correlations with pro-inflammatory markers, which were IFN-γ and IL-6 [33].

Another systematic review by Hamjane et al. assessed the association between gut microbiota dysbiosis, obesity, and its role in development of heart diseases and T2D [36]. The authors of the review noted that gut microbiota dysbiosis in obese patients is featured by a decrease in butyrate-producing bacteria [36]. This dysbiosis may potentially lead to production of a variety of metabolites, including branched-chain amino acids (BCAAs), lipopolysaccharide (LPS), short-chain fatty acids (SCFAs), bile acids, and imidazole propionate, all of these impact metabolism blood glucose. The review highlights that certain microbiota-derived metabolites, such as trimethylamine-N-oxide, SCFAs, and bile acids, are greatly linked with the development of CVDs [36]. The researcher summarized that gut microbiota dysbiosis is an important determinant in the development and process of these obesity-related metabolic abnormalities and signifies a critical target for dietary or pharmaceutical management [36].

A similar subsequent systematic review and meta-analysis was published by Mohammadi et al., in 2025, where authors noticed a strong relationship between circulating trimethylamine N-oxide (TMAO) levels and the likelihood of T2D [38]. The study, using 32 type 2 and gestational diabetes trials, found that diabetics have significantly greater levels of TMAO than non-diabetics [38]. It also demonstrated that elevated TMAO was associated with an increased odd of being diabetic [38]. This suggests that TMAO, a gut microbiota-derived metabolite, may play a significant role in the causation of the disease [38].

To provide a clearer understanding of the metabolic implications of microbial dysbiosis in T2D, Table 2 summarizes the major microbial taxa identified above in Section 3.1 and their associated metabolites. These metabolites, including short-chain fatty acids (SCFAs), lipopolysaccharides (LPS), bile acids, and trimethylamine N-oxide (TMAO), play pivotal roles in modulating insulin sensitivity, inflammation, and glucose homeostasis. Integrating microbial and metabolite data highlights how compositional shifts in gut microbiota contribute to the metabolic disturbances’ characteristic of T2D.

### 3.2. How Gut Bacteria Contribute to Type 2 Diabetes: Potential Mechanisms

#### 3.2.1. Bile Acids

Bile acids are another group of molecules that illustrate the influence of our gut microbes on our metabolism. When we eat, our liver produces primary bile acids, which are then modified by our gut microbiome into secondary bile acids [39,40,41]. These secondary bile acids play a role in the control of blood glucose through the stimulation of specific receptors in the gut, which in turn stimulates the secretion of GLP-1 [42,43,44]. This may explain why bariatric surgery, which alters gut anatomy and consequently reshapes the gut microbiome, often leads to improved glucose control and weight reduction, accompanied by increased bile acid levels that enhance metabolic signaling pathways involved in glucose and lipid metabolism [45]. Bile acids also function via another receptor, FXR, to reduce the glucose production in the liver, thereby improving the body’s ability to process blood sugar [46,47].

#### 3.2.2. Branched-Chain Amino Acids (BCAAs)

While some microbial metabolites are beneficial, others are not. Raised levels of the branched-chain amino acids (BCAAs) leucine, isoleucine, and valine have been linked to increased risk for T2D [48,49,50]. They are obtained from both the gut microbiome and diet, but excessive amounts of them can lead to insulin resistance [51,52]. Elevated BCAA levels have been found to worsen insulin signaling, making cells less responsive to insulin [53,54]. They also have the ability to suppress fat metabolism and make the liver release more sugar [54]. While we know BCAAs are involved in these pathways, additional work is needed to understand precisely how they act to promote the development of T2D [55].

#### 3.2.3. Lipopolysaccharides (LPS)

Lipopolysaccharides (LPS) are probably the most direct connection between gut dysbiosis and inflammatory process that occurs in the body. LPS is a component of the outer membrane of certain bacteria, and during gut dysbiosis, the gut barrier can become permeable, allowing LPS into the bloodstream [56]. This is frequent with high-fat diets, which promote the growth of LPS-producing bacteria [57]. Once in the blood, LPS acts as an alarm signal, triggering a vigorous inflammatory response in the body [58,59]. This chronic, low-grade inflammation is a major source of insulin resistance, resulting in impaired capacity of our cells to use insulin effectively [58,59]. Indeed, animal studies have demonstrated that providing LPS can lead to substantial rise in inflammatory markers and increased blood glucose levels, thus verifying its contribution in the development and progression of T2D [60,61].

#### 3.2.4. Short-Chain Fatty Acids (SCFAs)

SCFAs are a perfect example of how gut bacteria can be our friends. When we eat fiber, our gut microbes ferment it to produce SCFAs, namely butyrate, propionate, and acetate. These molecules are important for maintaining stable blood sugar. For instance, a study by Sanna et al. (2019) demonstrated that people with a genetic predisposition to produce more butyrate have better insulin responses [62]. Butyrate works by stimulating our intestinal cells to release hormones like glucagon-like peptide-1 (GLP-1) and peptide YY (PYY), which not only control appetite but also enhance insulin secretion and lower the release of sugar into the bloodstream [63]. Butyrate also keeps our gut lining intact by causing the release of tight-junction proteins, a sort of security fence that prevents unwelcome substances from passing into our bloodstream [64,65]. Propionate also helps with blood sugar control by also causing the release of GLP-1 and PYY and enabling our liver to make glucose in a controlled way [64,66]. In fact, clinical trials showed that propionate supplementation can reduce food intake and prevent the loss of insulin sensitivity that normally occurs with weight gain [67].

## 4. Discussion

The present narrative review synthesizes the emerging evidence on the gut microbiota–metabolic axis and its role in the pathogenesis and progression of type 2 diabetes mellitus (T2D). Across diverse populations and study designs, a consistent pattern emerges: T2D is associated with gut microbial dysbiosis, characterized by reduced diversity and a decrease in beneficial, butyrate-producing bacteria such as *Faecalibacterium prausnitzii*, *Roseburia intestinalis*, and *Eubacterium rectale*, alongside an enrichment of opportunistic pathogens and pro-inflammatory taxa including *Escherichia*-*Shigella*, *Lactobacillus*, and *Enterococcus*. These microbial alterations are not merely associative; they are functionally linked to metabolic dysregulation through multiple mechanisms. Specifically, dysbiosis contributes to chronic low-grade inflammation, impaired glucose metabolism, and insulin resistance, which collectively form the core pathophysiological processes in T2D.

**Figure 2:**
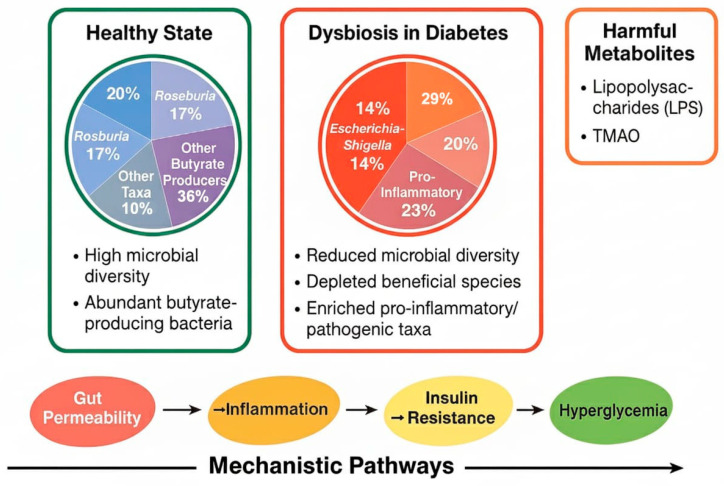
A schematic representation of gut microbiota alterations in type 2 diabetes.

A schematic representation of gut microbiota alterations in type 2 diabetes and their mechanistic links to metabolic dysfunction is shown in Figure 2. The schematic diagram illustrates the proposed link between gut microbiota dysbiosis and the pathogenesis of type 2 diabetes mellitus (T2D). In healthy individuals, a diverse microbial community dominated by butyrate-producing taxa such as *Faecalibacterium prausnitzii*, *Roseburia intestinalis*, and *Eubacterium rectale* maintains intestinal barrier integrity, supports insulin sensitivity, and suppresses inflammation through the production of short-chain fatty acids (SCFAs). In contrast, patients with T2D exhibit reduced microbial diversity and an overrepresentation of pro-inflammatory and opportunistic bacteria such as *Escherichia*-*Shigella* and *Enterococcus*. This imbalance leads to increased intestinal permeability, elevated lipopolysaccharide (LPS) levels, and systemic low-grade inflammation, ultimately contributing to insulin resistance and glucose dysregulation. The figure aligns with our review findings, emphasizing that these microbial alterations are functionally and mechanistically linked to the metabolic abnormalities characteristic of T2D, underscoring their potential as therapeutic targets.

The integration of microbiome composition and metabolite profile provides further insight into whether microbial alterations may contribute causally to diabetes development. As summarized in Table 2, the reduction in butyrate-producing taxa such as *Faecalibacterium prausnitzii* and *Roseburia intestinalis* corresponds with decreased levels of short-chain fatty acids (SCFAs), impairing insulin sensitivity and intestinal barrier function. Conversely, enrichment of *Escherichia*-*Shigella* and *Enterococcus* is associated with increased production of pro-inflammatory metabolites, including lipopolysaccharides (LPS) and branched-chain amino acids (BCAAs), which promote systemic inflammation and insulin resistance. This parallel shift in microbial structure and metabolite output supports the hypothesis that dysbiosis may play a contributory role in the pathogenesis of T2D rather than being a mere consequence of metabolic dysfunction.

However, an important question that remains is whether these microbiota changes are a cause or a consequence of diabetes. Most existing studies are cross-sectional, which limits causal inference. Evidence from emerging longitudinal and mechanistic studies suggests a bidirectional relationship, where metabolic dysfunction and hyperglycemia can reshape microbial composition, while dysbiosis may in turn exacerbate inflammation and insulin resistance. This highlights the need for well-controlled, prospective, and interventional studies to establish temporal causality and minimize bias, as microbiota alterations are observed across many diseases without necessarily being causative.

Emerging studies also highlight the role of microbial metabolites in mediating host metabolic responses. For example, short-chain fatty acids (SCFAs) produced by commensal bacteria enhance insulin sensitivity, modulate gut hormone secretion, and maintain intestinal barrier integrity. Conversely, elevated levels of trimethylamine N-oxide (TMAO), produced by microbial metabolism of dietary choline and L-carnitine, have been linked to increased risk of both T2D and gestational diabetes. Dysregulated bile acids, branched-chain amino acids (BCAAs), and lipopolysaccharides (LPS) further exemplify the complex interplay between gut microbes and host metabolic pathways. These findings collectively underscore the gut-metabolic axis as a potential target for therapeutic interventions, emphasizing the importance of integrating microbiome research into clinical practice.

### 4.1. Clinical Implications

From a clinical perspective, the implications of these findings are substantial. First, they support the rationale for microbiome-targeted interventions as adjuncts to conventional T2D therapies. Probiotics, prebiotics, synbiotics, and dietary interventions that increase SCFA-producing taxa may improve insulin sensitivity, reduce systemic inflammation, and aid in glycemic control. Early clinical studies suggest that supplementation with *Lactobacillus* and *Bifidobacterium* species can favorably alter gut microbial composition and improve metabolic outcomes. Additionally, fecal microbiota transplantation (FMT) has shown promise in pilot studies for enhancing insulin sensitivity, although standardized protocols and long-term safety data are still lacking.

Dietary modulation also represents a practical and widely applicable strategy. Diets rich in fiber, whole grains, fruits, and vegetables promote microbial diversity and the proliferation of beneficial bacteria, resulting in enhanced SCFA production and improved glucose metabolism. These findings reinforce current clinical guidelines recommending lifestyle modifications as the cornerstone of T2D management, while highlighting the microbiome as a mechanistic link underlying dietary benefits. Integration of microbiome profiling into clinical practice could enable precision nutrition and individualized interventions, where therapy is tailored to a patient’s specific microbial composition and metabolic status.

Moreover, the interplay between gut microbes and pharmacotherapy warrants consideration. Drugs such as metformin not only lower glucose but also modulate gut microbiota, enriching SCFA-producing taxa and potentially contributing to their therapeutic effects. Understanding these interactions can help optimize treatment regimens and predict inter-individual variability in drug response. Future research may also explore microbiota-directed pharmacotherapies, including microbial metabolite modulators and immunomodulatory approaches, to directly target pathogenic bacterial pathways.

### 4.2. Strengths and Limitations

This review has several strengths. By compiling findings from multiple study designs—including observational studies, metagenome-wide association studies, and systematic reviews—we provide a comprehensive synthesis of the current knowledge on the gut microbiota–T2D axis. The inclusion of studies across diverse populations enhances the generalizability of the observations and highlights consistent microbial patterns linked with T2D, despite geographical and methodological variability. Additionally, focusing on both taxonomic and functional aspects of the microbiome allows for an integrative perspective, emphasizing not only compositional changes but also their metabolic consequences. The narrative approach further enables the discussion of clinical relevance, translating microbial findings into potential therapeutic strategies. By incorporating mechanistic insights, metabolite profiles, and host-microbe interactions, this review offers a holistic understanding of how gut dysbiosis contributes to T2D pathogenesis and informs potential interventions.

Several limitations must be acknowledged. First, as a narrative review, this study does not provide quantitative synthesis or meta-analysis, which limits the ability to assess effect sizes or statistically compare outcomes across studies. Second, included studies exhibited heterogeneity in methodologies, such as variations in sequencing techniques, taxonomic resolution, and microbial profiling platforms, which may affect comparability. Third, many studies did not adequately control for confounding factors including diet, medication use, lifestyle, and comorbidities, which are known to influence gut microbial composition. Consequently, causality cannot be definitively inferred, and findings should be interpreted with caution. Generalizability may also be limited, particularly in populations underrepresented in existing studies, including those from low- and middle-income countries with unique dietary and environmental exposures. Finally, there is a risk of publication bias, as studies reporting significant microbial alterations are more likely to be published, potentially overestimating observed associations.

## 5. Conclusions and Future Directions

In summary, this review highlights the gut microbiota as a critical mediator of T2D pathogenesis and a promising target for therapeutic interventions. Dysbiosis, characterized by loss of beneficial taxa and expansion of pro-inflammatory microbes, contributes to chronic inflammation, insulin resistance, and glucose dysregulation. Clinical integration of microbiome knowledge, through dietary modulation, probiotics, and precision medicine approaches, may enhance glycemic control and overall metabolic health. While challenges remain regarding safety, efficacy, and generalizability, continued rigorous research will enable the development of personalized, microbiota-targeted strategies for T2D prevention and management. Harnessing the gut-metabolic axis may ultimately transform the clinical landscape of T2D, improving outcomes and quality of life for millions of affected individuals worldwide.

Future research should prioritize longitudinal, multi-ethnic, and large-scale studies to validate observed microbial patterns and clarify causal mechanisms. Personalized microbiome profiling may guide precision interventions, including tailored diets, probiotics, or pharmacotherapies. Investigating drug-microbiome interactions, long-term safety of microbiota-modifying interventions, and potential adverse effects (e.g., bacteremia, transfer of antibiotic resistance genes) remains essential. Additionally, emerging avenues such as microbiota-directed vaccines and gene-based therapies hold potential to revolutionize T2D management by directly targeting pathogenic microbes or host-microbial metabolic pathways.

## Figures and Tables

**Table 1:** Summary of Key Studies on Gut Microbiota Alterations Associated with Type 2 Diabetes Mellitus.

Study Author	Year	Setting	Study Design	Sample Size	Key Findings
Larsen et al. [29]	2010	Denmark	Observational study using real-time quantitative polymerase chain reaction (qPCR) and tag-encoded amplicon pyrosequencing of the V4 region of the 16S ribosomal RNA (rRNA) gene	36 male adults (18 T2D patients, 18 healthy controls)	Unfavorable microbiota shifts (increased in T2D): Opportunistic pathogens including *Bacteroides caccae*, *Clostridium hathewayi*, *Clostridium symbiosum*, *Eggerthella lenta*, *Clostridium ramosum*, and *Escherichia coli*; mucin-degrading species (*Akkermansia muciniphila*); sulfate-reducing bacteria (*Desulfovibrio* spp.); and *Betaproteobacteria* (associated with impaired glucose tolerance). Higher *Bacteroidetes*/*Firmicutes* and *Bacteroides*-*Prevotella*/*C. coccoides*-*E. rectale* ratios correlated positively with plasma glucose levels. Favorable microbiota shifts (reduced in T2D): Butyrate-producing bacteria including *Clostridiales* sp. SS3/4, *Eubacterium rectale*, *Faecalibacterium prausnitzii*, *Roseburia intestinalis*, and *Roseburia inulinivorans*, along with decreased abundance in *Firmicutes* and *Clostridia* class members.
Qin et al. [30]	2012	China (Chinese population)	Two-stage metagenome-wide association study (MGWAS) using deep shotgun sequencing of gut microbial deoxyribonucleic acid (DNA).	345 participants (145 T2D patients, 200 healthy controls)	Increased abundance in T2D: Opportunistic pathogens including *Bacteroides caccae*, *Clostridium hathewayi*, *Clostridium symbiosum*, *Eggerthella lenta*, *Clostridium ramosum*, and *Escherichia coli*; mucin-degrading bacteria (*Akkermansia muciniphila*); and sulfate-reducing species (*Desulfovibrio* spp.). Reduced abundance in T2D: Butyrate-producing bacteria including *Clostridiales* sp. SS3/4, *Eubacterium rectale*, *Faecalibacterium prausnitzii*, *Roseburia intestinalis*, and *Roseburia inulinivorans*. These compositional changes reflect a functional dysbiosis associated with altered gut metabolic activity and inflammation.
Karlsson et al. [31]	2013	Europe (European women; Swedish and Danish cohorts from the MetaHIT project)	Observational cohort study using metagenomic shotgun sequencing	145 women (53 T2D patients, 43 with impaired glucose tolerance, 49 with normal glucose tolerance)	Reduced abundance in T2D: *Roseburia intestinalis*, *Faecalibacterium prausnitzii*, and five *Clostridium* species. Increased abundance in T2D: Four *Lactobacillus* species. Positive associations: *Lactobacillus* species showed a direct correlation with fasting blood glucose and glycated hemoglobin (HbA1c) levels. Negative associations: *Clostridium* species were inversely correlated with fasting blood glucose, HbA1c, triglycerides, C-peptide, and serum insulin concentrations. Overall implication: Disruption in the balance between *Lactobacillus* and *Clostridium* species may contribute to T2D pathogenesis.
Forslund et al. [32]	2015	Multiple cohorts: China, Denmark (MetaHIT), Sweden	Meta-analysis of metagenomic data from prior studies, controlling for antidiabetic medication effects	Total 784 participants across cohorts: Chinese n = 256 (71 T2D with treatment info + 185 nondiabetic), Danish MetaHIT n = 383 (277 nondiabetic + 75 T2D + 31 T1D), Swedish n = 145 (53 T2D + 92 nondiabetic)	Effect of medication (Metformin): Confounds T2D gut microbiome results, enriching *Escherichia* species and reducing butyrate-producing taxa. Mechanistic insight: Metformin may improve glucose metabolism via short-chain fatty acid (SCFA) production. Post-metformin adjustment: Disease-specific microbiome signatures are revealed, notably a decrease in butyrate-producing bacteria. Overall conclusion: Distinguishing T2D-associated microbiota changes from drug effects is crucial for accurate characterization of dysbiosis.
Umirah et al. [33]	2021	Global (multiple populations)	Systematic review of 13 case–control studies	575 T2D patients and 840 healthy controls	Reduced abundance in T2D: Butyrate-producing bacteria, inversely associated with glycemic parameters, dominated in healthy controls. Increased abundance in T2D: *Lactobacillus* species, particularly associated with higher blood glucose levels. Inflammation-related findings: *Firmicutes* were positively correlated with inflammatory markers including interferon-gamma (IFN-γ) and interleukin-6 (IL-6). Effect of medication: Use of metformin and other drugs may confound associations between microbiota composition and T2D outcomes.
Letchumanan et al. [34]	2022	Global (multiple populations, not specified)	Systematic review of observational studies published from inception to February 2021	18 studies (5489 participants; prediabetes [preDM], newly diagnosed T2D [newDM], and normal glucose tolerance [nonDM])	Diversity: Lower gut microbial diversity in preDM and newDM participants compared with non-diabetic controls. Composition: Findings were inconsistent across studies. Trends in newly diagnosed T2D (n = 4 studies): Increased *Firmicutes* and decreased *Bacteroidetes*. Genus/species changes: Reduced *Faecalibacterium prausnitzii*, *Roseburia*, *Dialister*, *Flavonifractor*, *Alistipes*, *Haemophilus*, *Akkermansia muciniphila*; increased *Lactobacillus*, *Streptococcus*, *Escherichia*, *Veillonella*, *Collinsella*. Correlations: *Lactobacillus* positively associated with fasting plasma glucose, HbA1c, and/or homeostatic model assessment of insulin resistance (HOMA-IR) in four studies. Nutrition: Dietary factors influence bacterial abundances. Future directions: Further research is needed to clarify the role of *Lactobacillus* species and their consistent links with clinical biomarkers and nutrition.
Slouha et al. [35]	2024	Global (multiple populations)	Systematic review of observational studies	29 studies	Shared taxa (T2D vs. controls): *Bacteroides*, *Proteobacteria*, *Firmicutes*, *Actinobacteria* (with varying abundances). Decreased in T2D: *Lactobacillus* spp., *Faecalibacterium prausnitzii*—associated with insulin resistance. Increased in T2D: *Akkermansia muciniphila*—associated with high body mass index (BMI) and altered fat metabolism. Metabolites: Butyrates and melatonin implicated in T2D progression. Sex-specific findings: Low testosterone in T2D males correlated with higher abundance of *Gemella*, *Lachnospiraceae*, and *Massilia*. Knowledge gaps: Further studies are needed to clarify the effects of diet, exercise, and antidiabetic drugs on gut microbiota and glycemic control.
Hamjane et al. [36]	2024	Global (multiple populations)	Systematic review	>150 articles	Decreased in T2D: Butyrate-producing bacteria. Dysbiosis-driven metabolites affecting glucose metabolism: Short-chain fatty acids (SCFAs), bile acids, lipopolysaccharides (LPS), branched-chain amino acids (BCAAs), and imidazole propionate. Overall conclusion: Alterations in these metabolites contribute to the development and progression of T2D
Chong et al. [37]	2025	Global (multiple populations)	Systematic review of observational studies published between 2010 and 2024	58 studies	Beta diversity: Differed significantly between T2D patients and controls. Positively correlated with T2D: *Lactobacillus*, *Escherichia*-*Shigella*, *Enterococcus*, *Subdoligranulum*, *Fusobacteria*. Negatively correlated with T2D: *Akkermansia*, *Bifidobacterium*, *Bacteroides*, *Roseburia*, *Faecalibacterium*, *Prevotella*. Consistent associations: *Escherichia*-*Shigella* showed a positive relationship with T2D. Protective species: *Faecalibacterium prausnitzii*.
Mohammadi et al. [38]	2025	Global (multiple populations)	Systematic review and meta-analysis	32 studies	Metabolite findings: Trimethylamine N-oxide (TMAO) levels were substantially higher in T2D patients compared with controls. Health implications: Elevated TMAO levels were associated with increased risk of both T2D and gestational diabetes mellitus (GDM).

T2D: Type 2 diabetes mellitus; MGWAS: Metagenome-wide association study; DNA: Deoxyribonucleic acid; spp.: Species pluralis (multiple species of a genus); qPCR: Quantitative polymerase chain reaction; rRNA: Ribosomal ribonucleic acid; HbA1c: Glycated hemoglobin; MetaHIT: Metagenomics of the Human Intestinal Tract project; T1D: Type 1 diabetes mellitus; SCFA: Short-chain fatty acids; IFN-γ: Interferon-gamma; IL-6: Interleukin-6; preDM: Pre-diabetes; newDM: Newly diagnosed T2D; nonDM: Non-diabetic; HOMA-IR: Homeostatic model assessment of insulin resistance; BMI: Body mass index; LPS: Lipopolysaccharides; BCAAs: Branched-chain amino acids; TMAO: Trimethylamine N-oxide; GDM: Gestational diabetes mellitus.

**Table 2:** Key gut microbial taxa in T2D and their associated metabolites and metabolic effects.

Microbial Taxa	Primary Metabolite(s)	Effect on Host Metabolism	Association with T2D
*Faecalibacterium prausnitzii*	Butyrate (SCFA)	Enhances insulin sensitivity, reduces inflammation, maintains gut barrier integrity	↓ Decreased in T2D
*Roseburia intestinalis*	Butyrate (SCFA)	Anti-inflammatory; improves glucose metabolism	↓ Decreased in T2D
*Eubacterium rectale*	Butyrate (SCFA)	Promotes GLP-1 secretion, supports metabolic balance	↓ Decreased in T2D
*Akkermansia muciniphila*	Mucin degradation; acetate, propionate	Improves gut barrier, metabolic regulation	Mixed (↑ in some T2D; ↓ in others)
*Lactobacillus* spp.	Lactic acid, acetate	Strain-dependent: some improve metabolism, others correlate with hyperglycemia	↑ Increased in T2D
*Escherichia*-*Shigella*	Lipopolysaccharides (LPS)	Induces inflammation, increases gut permeability	↑ Increased in T2D
*Bacteroides* spp.	TMAO, secondary bile acids	Alters lipid and glucose metabolism	↑ Increased in T2D
*Clostridium* spp.	Butyrate, LPS (strain-dependent)	Some are protective (butyrate-producing), others pathogenic	Mixed effects
*Desulfovibrio* spp.	Hydrogen sulfide (H_2_S)	Impairs gut barrier; promotes inflammation	↑ Increased in T2D
*Eggerthella lenta*	Phenolic metabolites	May modulate drug metabolism and inflammation	↑ Increased in T2D

↓: Decreased; ↑: Increased.

## Data Availability

No new data generated for this article. The dataset(s) supporting the conclusions of this article is included within the article.

## Supplementary Materials

The following supporting information can be downloaded at: https://www.mdpi.com/article/10.3390/medicina61112017/s1, Table S1: Scale for the Assessment of Narrative Review Articles-SANRA.

## Abbreviations

The following abbreviations are used in this manuscript:

T2D	Type 2 diabetes mellitus
T1D	Type 1 diabetes mellitus
GDM	Gestational diabetes mellitus
preDM	Pre-diabetes
newDM	Newly diagnosed T2D
nonDM	Non-diabetic
BMI	Body mass index
SCFA	Short-chain fatty acids
TMAO	Trimethylamine N-oxide
BCAAs	Branched-chain amino acids
LPS	Lipopolysaccharides
IFN-γ	Interferon-gamma
IL-6	Interleukin-6
HbA1c	Glycated hemoglobin
HOMA-IR	Homeostatic model assessment of insulin resistance
MGWAS	Metagenome-wide association study
qPCR	Quantitative polymerase chain reaction
rRNA	Ribosomal ribonucleic acid
DNA	Deoxyribonucleic acid
MetaHIT	Metagenomics of the Human Intestinal Tract project
FMT	Fecal microbiota transplantation
