# Supplementary material for: The Gut Microbiota–Metabolic Axis: Emerging Insights from Human and Experimental Studies on Type 2 Diabetes Mellitus—A Narrative Review
Source: Medicina (Kaunas). 2025 Nov 11;61(11):2017. doi: 10.3390/medicina61112017 (PMC12654423; doi:10.3390/medicina61112017)
Supplement: Supplementary file 1 [file medicina-61-02017-s001.zip › medicina-3941311-supplementary.pdf]

medicina

MDPI

Supplemental Table S1: Scale for the Assessment of Narrative Review Articles - SANRA			
**Category**	**Option**	**Score**	**[Check Box]**
1) Justification of the article's importance for the readership	The importance is not justified.	0	[ ]
	The importance is alluded to, but not explicitly justified.	1	[ ]
	The importance is explicitly justified.	2	[√]
2) Statement of concrete aims or formulation of questions	No aims or questions are formulated.	0	[ ]
	Aims are formulated generally but not concretely or in terms of clear questions.	1	[ ]
	One or more concrete aims or questions are formulated.	2	[√]
3) Description of the literature search	The search strategy is not presented.	0	[ ]
	The literature search is described briefly.	1	[ ]
	The literature search is described in detail, including search terms and inclusion criteria.	2	[√]
4) Referencing	Key statements are not supported by references.	0	[ ]
	The referencing of key statements is inconsistent.	1	[ ]
	Key statements are supported by references.	2	[√]
5) Scientific reasoning	(e.g., incorporation of appropriate evidence, such as RCTs in clinical medicine)		
	The article's point is not based on appropriate arguments.	0	[ ]
	Appropriate evidence is introduced selectively.	1	[ ]
	Appropriate evidence is generally present.	2	[√]
6) Appropriate presentation of data	(e.g., absolute vs relative risk; effect sizes without confidence intervals)		

	Data are presented inadequately.	0	[ ]
	Data are often not presented in the most appropriate way.	1	[ ]
	Relevant outcome data are generally presented appropriately.	2	[✓]
